# Shielded Cone Coil Array for Non-Invasive Deep Brain Magnetic Stimulation

**DOI:** 10.3390/bios14010032

**Published:** 2024-01-09

**Authors:** Rawan Abu Yosef, Kamel Sultan, Ahmed Toaha Mobashsher, Firuz Zare, Paul C. Mills, Amin Abbosh

**Affiliations:** 1 The School of Electrical Engineering and Computer Science, The University of Queensland, St. Lucia, QLD 4072, Australia; r.abuyosef@uq.edu.au (R.A.Y.); f.zare@qut.edu.au (F.Z.); a.abbosh@uq.edu.au (A.A.); 2 The School of Veterinary Science, The University of Queensland, Gatton, QLD 4343, Australia; p.mills@uq.edu.au

**Keywords:** transcranial magnetic stimulation, deep brain stimulation, neurological disease, coil array, vivo pig, Alzheimer

## Abstract

Non-invasive deep brain stimulation using transcranial magnetic stimulation is a promising technique for treating several neurological disorders, such as Alzheimer’s and Parkinson’s diseases. However, the currently used coils do not demonstrate the required stimulation performance in deep regions of the brain, such as the hippocampus, due to the rapid decay of the field inside the head. This study proposes an array that uses the cone coil method for deep stimulation. This study investigates the impact of magnetic core and shielding on field strength, focality, decay rate, and safety. The coil’s size and shape effects on the electric field distribution in deep brain areas are also examined. The finite element method is used to calculate the induced electric field in a realistic human head model. The simulation results indicate that the magnetic core and shielding increase the electric field intensity and enhance focality but do not improve the field decay rate. However, the decay rate can be reduced by increasing the coil size at the expense of focality. By adopting an optimum cone structure, the proposed five-coil array reduces the electric field attenuation rate to reach the stimulation threshold in deep regions while keeping all other regions within safety limits. In vitro and in vivo experimental results using a head phantom and a dead pig’s head validate the simulated results and confirm that the proposed design is a reliable and efficient candidate for non-invasive deep brain magnetic stimulation.

## 1. Introduction

Many progressive neurological diseases like Alzheimer’s disease initially affect the hippocampal region of the brain. Unlike old hypotheses that held that the adult brain is a rigid structure, recent research has demonstrated that the brain can change even through adulthood [1,2,3,4]. The ability of the brain to reorganize itself in response to inputs and experience by generating new neural connections is referred to as neuroplasticity. It can be seen in a variety of brain reactions, including cortical remapping in response to injury and brain healing after stroke. Furthermore, there is evidence that neurogenesis (the formation of new brain cells) occurs in old age, although this evidence is largely restricted to the hippocampus [1]. The hypothesis of neuroplasticity offers promise for reversing Alzheimer’s disease by stimulating the damaged parts of the brain, particularly the hippocampus, in preclinical phases.

Transcranial magnetic stimulation (TMS) is a non-invasive technique used to stimulate the brain by generating a magnetic field through a coil. This field produces an electric field across the neurons in the brain which then creates eddy currents. When the electric field exceeds a certain threshold, it triggers the action potential—the basic signal produced by neurons. The signal is then propagated through neural pathways, leading to stimulation [5].

One of the main challenges with TMS systems is the rapid decay of the electric field induced by the TMS coil. As a result, to stimulate a deep region, the concentration of a much higher electric field in the cortical regions is required, which can cause discomfort or pain for the patient. In some cases, this can even exceed the suggested safety guidelines for that cortical region [6,7], leading to further complications such as seizures.

The decay rate of the electric field depends on the coil’s geometry. Therefore, many researchers have investigated better coil geometries for deep brain stimulation since the technology was introduced [8,9,10,11,12,13]. Since the first TMS system was demonstrated [5], the design of the coil used to stimulate the brain has received considerable attention for refining two main characteristics of the TMS-induced electric field: focality and the depth of penetration. Changing the shape and structure of the coil alters the features of the magnetic field it generates, which alters the distribution of the electric field created in the brain. It was discovered that by adjusting the coil design, the stimulation focality and the depth of penetration can be controlled. There are now numerous coil designs that differ in terms of stimulation depth and focality. However, the trade-off between focality and depth appears to persist.

Various optimization schemes were proposed to increase the field focality of circular coils, such as adjusting the size, number of turns, or concavity of the coil [14,15]. Other efforts have been made to increase the electric field induced in the brain by modifying coil configurations, such as the figure-of-eight coil [16], which consists of two adjacent loops with alternating current directions. Many other coils then began to emerge, like the butterfly coil and the 3D differential coil [17]. Additionally, conductive shielding plates were added to improve the TMS-induced electric field accumulation degree [18,19].

From the perspective of increasing the depth of penetration, a significant number of researchers also proposed new coil designs. For instance, the double-cone coil consists of two adjacent circular coils attached at a specific angle [20]. Also, using a ferromagnetic core with high permeability, like the proposed enhanced Hesed coil, increases the skin depth [21]. Moreover, several attempts were made to stimulate deep brain regions, like with the large halo coil and the high 3D resolution coil [11,22]. Coil arrays have also been studied to improve the flexibility of the stimulation and control the distribution of the induced electric field [23,24].

Other efforts include modifying the coil by adding a ferromagnetic structure such as a core or shield. Several studies have demonstrated that adding a high-permeability core can enhance the decay rate of the TMS coil [25,26,27,28]. Another structure that has been researched to increase the safety and focality of the TMS system is ferromagnetic shielding. Adding a shield around a TMS coil can improve safety for the operator [29,30] and enhance the focality of TMS fields, as shown in [19,31,32]. 

In a previous study conducted by the authors of this paper [33], the effects of adding a ferromagnetic core and shield to a proposed cone coil design on the induced electric field intensity, focality, and decay rate were investigated. A parametric study was also conducted to evaluate the impact of coil size on these parameters. It was shown that employing a single coil to activate a deep brain area safely is challenging. In agreement with guidelines on the safety of repetitive transcranial magnetic stimulation (rTMS), the maximum safe intensity of 220% of the threshold value, Eth, was chosen as a safety limit, while other parameters like duration and repetition frequency were controlled [6].

Deep brain stimulation is an essential form of neuromodulation that is delivered to patients invasively [34]. In this paper, a non-invasive deep brain stimulation approach using an array of TMS coils is proposed. With a wise choice of the coils’ position, orientation, tilt, and size and the pulse frequency that affects the E field threshold value, the coil array configuration can reduce the field in superficial regions concerning a deep targeted area efficiently. 

Various coil array configurations are reported in the literature [35,36,37,38,39,40], mainly the multi-channel configuration of magnetic field generators capable of producing focused magnetic field (B field) patterns by serving as a highly directed near-field array [41]. It has been shown that near-field arrays may create desirable field patterns [36,37]. However, no actual array was developed to produce arbitrary field patterns due to constraints in the number of elements and existing processing [35]. Ruohonen et al. [42] introduced the multi-channel coil array in 1998. A multi-channel coil array comprises numerous coils that are arranged based on the space arrangement rule. 

Previous multi-coil magnetic stimulation devices were designed based on basic coil shapes such as planar and hemispherical layouts. Furthermore, these coils were tested on basic models like free-space or random conductors to measure and analyze the electric field inside conductive tissues [23,37,41,42,43,44]. However, to the best of our knowledge, no research has been conducted on examining a coil array for deep brain stimulation based on realistic simulations and experiments. 

This paper presents a new system for deep brain stimulation that uses a multi-coil array. The system is based on a cone coil with dimensions determined by the lowest decay rate. Various coil array geometries are constructed and evaluated using finite element method (FEM) simulations. This study investigates the optimal number of coils and their orientation by comparing the ratio of the maximal superficial E field on the grey matter to the E field induced at the targeted area, the hippocampus (hippo). This ratio (Egm/Ehippo) should be less than 2.2 to meet the maximum safe intensity. To achieve this safety ratio, a coil array system with five coils positioned in a relatively tangential orientation around the head is presented. Numerical studies show that this system significantly improves the electric field attenuation rate when mounted on a realistic head phantom, and achieves the safety ratio within the guidelines, making it a feasible option for non-invasive deep TMS. The experimental validation of the system on fresh dead pig and head phantom confirms agreement in the electric field distribution between simulations and measurements. 

## 2. System Conceptual and Design Principle 

### 2.1. Modeling and Simulation 

A realistic simulation environment is created using the FEM-based electromagnetic tool Sim4life to calculate electric and magnetic fields in different regions of the brain [45]. To that end, a multimodal imaging-based detailed anatomical (MIDA) model, which is a three-dimensional high-resolution computer-aided design model of the head and neck [46], is utilized in this study. All the tissues are associated with their frequency-dispersive dielectric properties.

The simulation uses two Sim4life solvers; the low-frequency magnetostatic vector potential solver is used to compute the B field, while the overall field acquired from this solver is utilized in the quasistatic low-frequency solver to estimate the E field inside a head model. The former solver offers the flexibility to handle varied permeability across the domain, which is essential for precisely gauging the iron core and shielding effects on the fields. In the simulator, the core and shield for the TMS coils are modeled as a homogeneous material with a relative permeability of 5000 [28]. The current waveform was modeled as sinusoidal with a 5 kHz frequency and a peak value of 100 A.

### 2.2. The Structural Design of the Cone Coil

**Figure 1:**
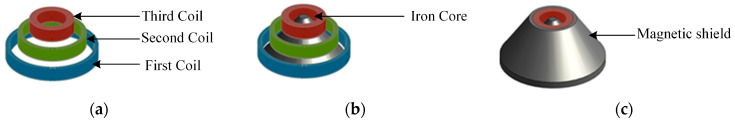
The proposed coil geometry: (**a**) the coil configuration, (**b**) three coils with an iron core, and (**c**) the final shape after adding a magnetic shield.

**Figure 2:**
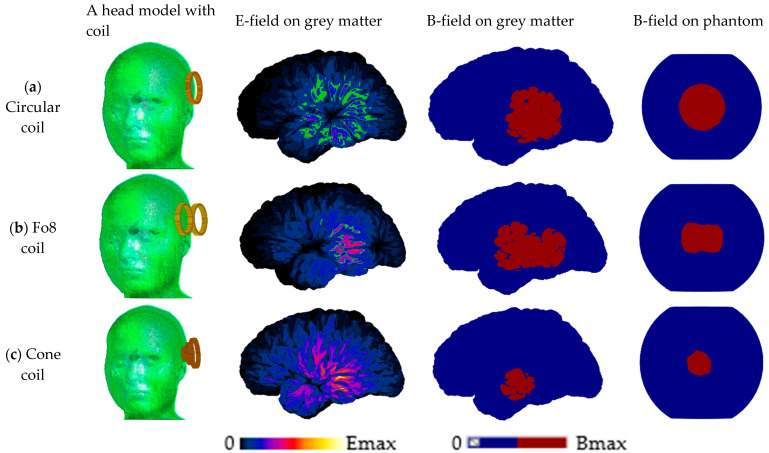
TMS performance with different configurations of coils on the MIDA head model and an equivalent phantom. In the second column, the E field distribution is highlighted in the region with a minimum value of Emax/2 area, while the B field of minimum value of Bmax/2 is highlighted in red in the third and fourth columns.

The cone coil, which was presented by the authors of [33], is utilized in this study. This coil, as opposed to traditional one-layer TMS coils, employs three concentric paired coils in three layers, as shown in Figure 1, and the details of the coil are listed in Table 1. The current supply is connected to the large element coil in the rear, which is connected to the medium coil in the middle layer and the small coil in the top layer, near the human head. The large coil, which is placed distant from the scalp, slows the attenuation of the field inside the skull because the E-field impinging on the skull is more diffuse and decays more slowly with longitudinal displacement from the coil (i.e., achieving better penetration depth). The small coil concentrates the fields in the desired direction and location of the head. Figure 2 displays the induced E field strength and focality at the grey matter utilizing the proposed and classic circular and figure-of-eight (Fo8) coils, respectively. The E field value and distribution in the second column show that adopting the cone coil enhances field intensity over circular and Fo8 coils. Furthermore, when comparing the field focality [47], which is defined as the area bounded by 50% of the peak E or B (E>Emax/2 and B>Bmax/2) of the three coils, the proposed coil outperforms the others, as shown in Figure 2.

To study the effect of adding a ferromagnetic structure on a coil’s performance, simulations were performed with and without modifications on two different coils, and the results were published in a previous study by the authors of this article [33]. The E field value and distribution on the grey matter demonstrate the modified coil’s improvements in field strength and focality [33]. Using a core improves the field value by 95%, and adding shielding boosts the improvement to 120%. Hence, the proposed coil is modified by adding a ferromagnetic core and shield structures. The high permittivity of the iron core aids in increasing and focusing the magnetic field at the coil’s center. The core has a conical form that fits in the coil and creates a concentrated and more powerful magnetic field on its tip near the head surface (see Figure 1). On the other hand, a cylindrical core can be used with the coil to minimize the net weight of the TMS system; it is also cheaper and easier to fabricate. Additionally, a conical ferromagnetic shield is placed around the coil so the magnetic flux passes easily through it, preventing it from leaking out. This strategy aims to increase clinician safety, enhance focality, increase stimulation intensity, and eliminate mutual coupling between adjacent coils [33].

**Figure 3:**
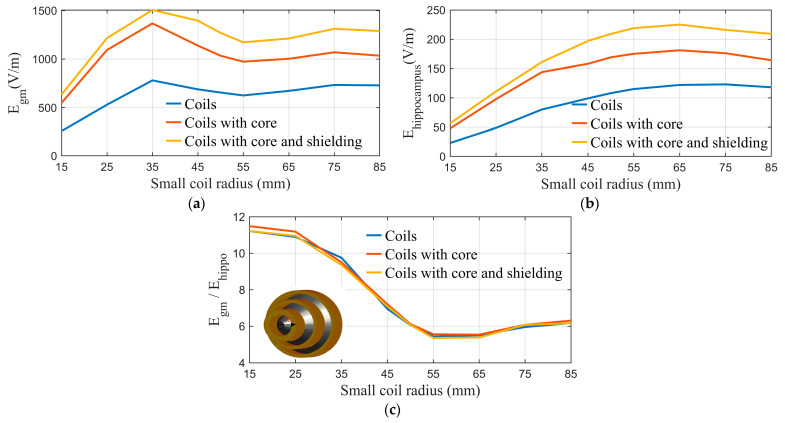
Performance evaluation of cone coil: (**a**) maximum E field at the surface of the grey matter vs. R; (**b**) maximum E field at the surface of the hippocampus vs. R. (**c**) Egm(max)/Ehippo(max) ratio vs. R.

A parametric study is performed on the coil, assuming different dimensions to investigate the effect of its size on field intensity and the decay rate. In each case, the maximum values of the field on the head phantom, the surface of the grey matter, and the surface of the hippocampus are calculated. Figure 3a,b demonstrate the relation between coil size and the maximum field values on the grey matter and hippocampus, respectively. Figure 3c depicts the ratio of the maximum field values at the grey matter and hippocampus (Egm(max)/Ehippo(max)) to examine the influence of coil size on the decay rate.

The high-permeability ferromagnetic structure improves field intensity and focality, allowing a potentially lower current to be used and reducing the stimulator’s burden. However, this highlights the issue of field rapid decay (as shown in [33]). The fundamental difficulty with TMS-based deep brain stimulation is the rapid decay of the induced electric field with depth. To achieve the threshold value in the deep brain area, a substantially larger electric field value will be centered on the head surface and cortical regions, raising safety concerns.

This issue can be addressed by increasing the coil size, as indicated by the parametric study conducted. While the strength of the E field is proportional to the size of the coil, as shown in Figure 3a,b, the ratio of the E field between the grey matter and the hippocampus is inversely related to the size of the coil, as shown in Figure 3c, indicating an improvement in the decay rate.

## 3. Design of the Coil Array

To activate deep regions of the brain, such as the hippocampus, a coil array is proposed to lower the field at cortical areas compared to deep targeted regions. This section illustrates different coil array designs to select the optimum coil number and arrangement for deep brain stimulation.

### 3.1. Number, Orientation, and Current Direction of the Coil 

As mentioned in Section 2.2, coil size significantly impacts the field’s decay rate; larger coils decay at a slower rate. The electric field focality and decay rate are also affected by the coil’s geometry. The cone coil is found to generate better decay rates without impacting the system’s focality. In this section, a coil with dimensions that indicate the best decay rate, as shown in Figure 3c, is utilized as a primary coil in the array. Although it is recommended to utilize a ferromagnetic shield in conjunction with the coils to prevent undesired mutual coupling, this practice affects the field decay rate, as shown in [33], and adds weight and expense to the device. Furthermore, the field created by the shielded coil is more focused than the field generated by the unshielded coil, which limits the cancellation of magnetic fields from nearby coils, which is the primary reason for lowering the field in the superficial areas. As a result, the developed coil array employs an unshielded coil.

After testing a variety of numbers and alignments of coils, the optimum results are obtained when the coils are placed tangentially to the head surface. The orientation of the coil elements relative to brain tissue dramatically affects the intensity and decay rate of the electric field throughout the brain. Since brain tissue is conductive and the air and skull are nearly insulators, the vector potential will create a buildup of electrical charge at the brain surface unless the induced electrical field is tangential to it. Many studies have confirmed that coil components perpendicular to the head surface cause surface charge accumulation, resulting in the full cancellation of the perpendicular component of the generated field within the tissue and a significant reduction in any other direction [10,48,49,50].

In multi-coil magnetic brain stimulation, the stimulating field may be formed and targeted by suitably altering the number of coils and driving currents. The induced electric field generated by employing multiple coils is a superposition of the fields from each coil. The partial cancellation of fields from individual coils enhances field focality and reduces the cortical field relative to the deep region [41].

**Figure 4:**
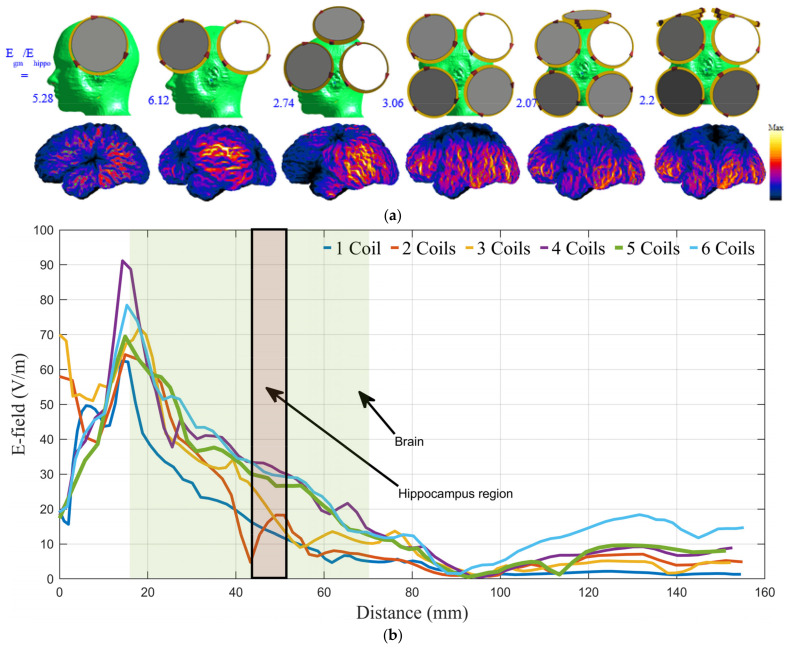
(**a**) The E field distribution on the grey matter surface achieved using various coil array geometries (R). (**b**) The distribution of the E field on a test line passing through the coronal cross-section of the phantom is shown inside the figure for the six-coil array geometries in (**a**).

The number of array coils and current direction are explored by calculating the safety ratio (Egm/Ehippo) in various scenarios of different numbers of coils and current directions, as shown in Figure 4a. The array with five coils positioned in a relatively tangential orientation around the head has the best (Egm/Ehippo) ratio of 2.07, satisfying safety limits (less than 2.2). Figure 4a illustrates the E-field distribution on the surface of the grey matter for each example and the ratio obtained from the various coil array configurations. Also, the distribution of the E-field on a test line passing through the coronal cross-section of the phantom (see Figure 4b) for the six-coil array geometries investigated in Figure 3a are obtained and illustrated in Figure 4b. The five-coil array configuration offers the slowest attenuation rate. As a result, the five-coil array configuration can be used to stimulate the deep brain while abiding by safety restrictions at other cortical regions of the brain.

**Figure 5:**
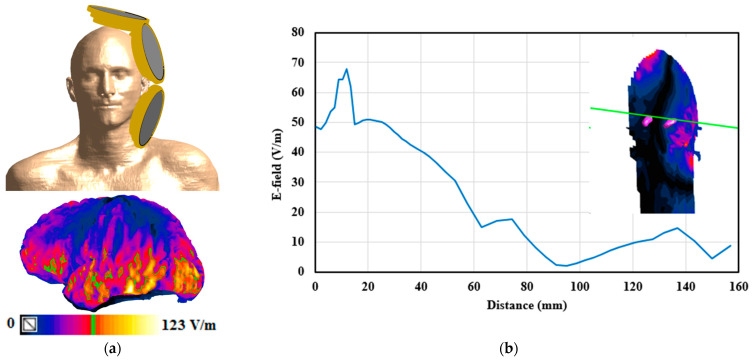
(**a**) The coil array system mounted on the Duke head model and the obtained E-field distribution on the grey matter with Emax/2 highlighted in green in the top and bottom images, respectively. (**b**) The E-field distribution along a test line that passes through the coronal head slice is shown in the inset.

The design is tested on another subject to examine if the gains are consistent across participants; Duke, a member of the IT’IS virtual population, is picked as another phantom for testing the coil array [51]. Duke is a high-resolution, accurate, whole-body anatomical virtual human model of a 34-year-old adult male. Duke’s head is distinct in geometry and size from MIDA’s; Figure 5a illustrates the head of the Duke phantom with the coil array system and the distribution of the induced E field on the grey matter in the top and bottom images, respectively. The grey matter and hippocampus have maximum E field values of 123 V/m and 58 V/m, respectively; the safety ratio is 2.12, which is within the recommended limits. 

Additionally, the E-field distribution along a test line that traverses a coronal head slice is obtained and shown in Figure 5b. Despite differences in the heads of the two phantoms, Duke and MIDA, their resulting E-field distributions, E-field maximum values, decay rates, and safety ratios exhibit similarities with minor differences. With both phantoms, a modest change in the positioning of the coil array while preserving the coil order and current direction results in a minor divergence in the field, but the safety ratios stays between 1.91 and 2.17, which is below the guideline limits.

### 3.2. Coil Array System Optimization

Many factors, such as system flexibility, weight, cost, safety, and power consumption, should be addressed for design optimization. To test the flexibility of the coil array system, safety inside the brain when a semi-random tangential orientation of the five coils is employed on the head phantom is evaluated. The results indicate that when the system coils are arranged tangentially to the head surface while considering their location and current direction, it is flexible and safe to use even with diverse head sizes and shapes.

More power is required as the number of coils increases because focusing includes the partial cancellation of magnetic fields from adjacent coils. Yet the increase in power is closely related to coil size. According to [41], the power escalation induced by increasing the number of coils is greatly reduced for larger coils. To reduce power dissipation, the proposed coil array system compensates for the increase in the coil number with an expansion in coil size, as mentioned in Section 3.1. 

**Figure 6:**
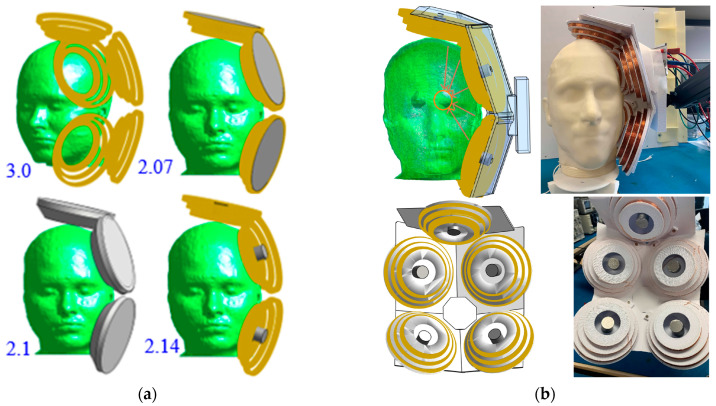
(**a**) The five-coil array with and without ferromagnetic structures, and the adapted geometry for experimental testing in the top and bottom rows, respectively (the values illustrated next to the head phantom are the ratio between the maximum E fields at the grey matter and the hippocampus). (**b**) The fabricated coil array.

Figure 6a shows that the desired safety ratio between the grey matter and hippocampus E fields can be attained with or without shielding, and by using either a cone or a cylindrical core. Although the lowest ratio is associated with the cone core, the cylindrical core is selected for fabrication and testing due to its easy fabrication, affordability, and light weight. Although this strategy restricts the advantage of the core in field improvement, it nevertheless passes safety regulations and minimizes the total weight of the TMS system.

## 4. System Validation

### 4.1. Coil Array Fabrication

To fabricate the array, five coils are made from 0.7 mm of enameled copper wire which is coiled around a 3D-printed model. The coils are then mounted on a 3D-printed stand that is designed to fix the coils’ position and orientation in the desired configuration, as shown in Figure 6b. The coil terminals are soldered to a board with plugs to easily connect the coils in series or parallel with the pulse generator. A cylindrical core with a diameter of 25 cm and a relative permeability of 1400 at 0.8 Tesla, which produces a saturation flux density of 2.2 Tesla, is used in conjunction with the coil. Finally, for easy installation of the coil array on the testing phantoms, the coil stand is attached to a handle that can be flexibly moved in three directions. The constructed coil array is depicted in Figure 6b.

### 4.2. Experimental Setup

The experimental tests must evaluate two essential points: first, the simulation results. Second, the coil array is compared to a single coil. For that purpose, the electrical field value and distribution of the cone coil and coil array are assessed on both a head phantom and on a fresh dead pig and compared with the in silico numerical simulation.

**Figure 7:**
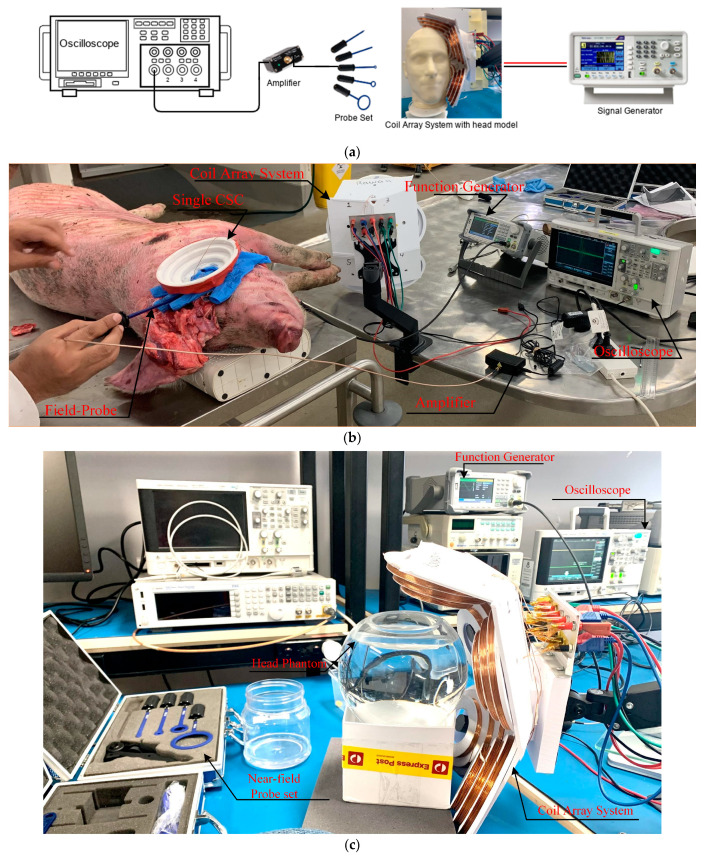
(**a**) Schematic diagram of the proposed system, (**b**) experimental setup for postmortem pig tests, and (**c**) experimental setup on liquid head phantom.

A spherical human head model (15 cm diameter) filled with a physiologic saline solution is used for lab investigations, while the pig head measurements are carried out on a dead pig’s head (postmortem), (see Figure 7). All the measurements were obtained within a few hours of the pig’s death. Since the induced voltage directly reflects the electromagnetic field, as demonstrated by Faraday’s law, experimental testing is carried out by passing pulses from a function generator (SIGLENT Technologies (SDG6000X series)) through the coil and measuring the induced voltage inside the head models with the probe. The function generator used to deliver the stimulation can generate waveforms with a maximum voltage of 20 Vpp and a maximum bandwidth of 50 MHz. A pulse waveform of 20 Vpp and a frequency of 5 kHz are utilized to excite the coils and obtain a clear output from the probe. The field inside the head phantom is measured using three distinct probes: a two-wire dipole probe with 1.5 cm between the two tips, a simple sensing coil with a diameter of 2 cm, and an RF Near Field Probe Set (Aaronia AG). All probes are covered with an insulating layer, thus allowing for safe measurements. The probes are moved in three directions within the head model and pig head with a 1 cm resolution displacement system, and the field distribution is measured in the entire head model volume with 1–2 cm precision. The probe is connected to a digital storage oscilloscope (Keysight Technologies InfiniiVision DSO-X 2002A) through a high gain low-frequency pre-amplifier (Aaronia AG), and the electrical field is calculated from the measured maximal voltage. The coils in the experimental setup are positioned on the left side of each head model to mimic the simulation, as shown in Figure 7. The experimental setup schematic diagram, postmortem experimental setup, and in vitro experimental setup are depicted in Figure 7a–c, respectively.

### 4.3. In Vitro Validation

For in vitro validation, the electric fields from the simulation and experiments of the cone coil and coil array applied on a homogeneous spherical model of the head are obtained and assessed. Although the spherical model simplifies head geometry, it is a practical reduction for studies that focus on the electric field characteristics of TMS coils. Furthermore, the results obtained with the spherical model are not limited to a particular subject’s head anatomy and coil position, allowing for general conclusions about the effects of coil properties and stimuli. The spherical model also provides a standard framework for studying coil configurations that can be easily reproduced by other researchers [52,53,54,55].

A homogeneous sphere with a diameter of 15 cm and an anisotropic conductivity of 0.33 S/m is used to model the human head in the Sim4life simulation environment. Since the magnetically induced electric field in a sphere is insensitive to radial variations in conductivity [54,56], the individual head tissue layers are not distinguished. The cone coil and the coil array are applied to the phantom.

**Figure 8:**
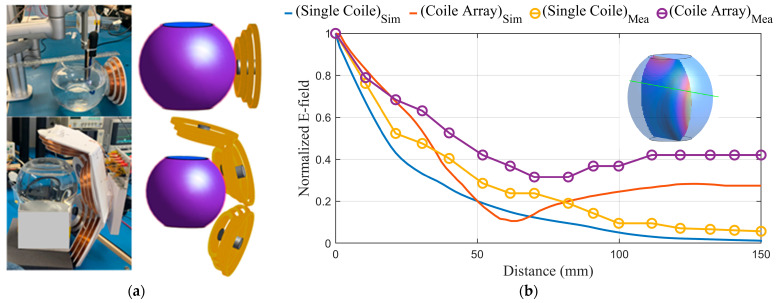
(**a**) The spherical head model with one cone coil and with the coil array in the simulation and measurements. (**b**) The simulation and measurement results for the E field value on a test line passing through the spherical phantom with one coil and with the coil array.

The in vitro experimental model also consists of a spherical glass tank with a diameter of 15 cm, as shown in Figure 8a. The tank is filled up to a liquid height of 12–14 cm. This model emulates the brain as a homogeneous conductor with an average brain conductivity of 0.33 S/m, obtained using a 0.2% saline solution [57]. Figure 8b depicts the simulation and measurement results for the normalized E field value on a test line subjected to a single coil and the coil array system.

### 4.4. Validation on Postmortem Tissue

While in vitro studies are useful for validating the simulation results of the coil array design, assessing its performance, and comparing it to a single coil, the effect of tissue conductivity variation is not included. Scientific studies over the last five decades have demonstrated that pigs have a high potential for biomedical research due to biological similarities to the human body and the pig’s suitability as a laboratory animal [58,59,60,61,62,63]. Since the dielectric properties of pig brain tissues are similar to those of humans [64,65], postmortem validation is performed on cadaver pig heads as analogs for human heads. The postmortem validation is carried out in accordance with ethical approval from the University of Queensland Animal Ethics Committee (221/AE000776).

Within a few hours of its death, a dead pig’s head is utilized to test the coil array system. The pig, a 10-month-old Danish Landrace sow (female) weighing ~95 kg, had no history of head injuries. To facilitate the measurement of the electric field within the skull, a boning knife is used to cut into skin and tissue around the skull enclosing the brain from the upper left side, and then a Bosch GOP 10 saw is used to cut through the skull, as illustrated in the bottom row of Figure 6a. The pig head is then fitted with the coil array system and a single cone coil separately, and the electric and magnetic fields inside the brain tissues are measured using the near-field probes. Inside the pig brain, the near-field probes are moved in three directions, and the field distribution of each coil system is measured in the entire brain volume with a 1 cm resolution.

**Figure 9:**
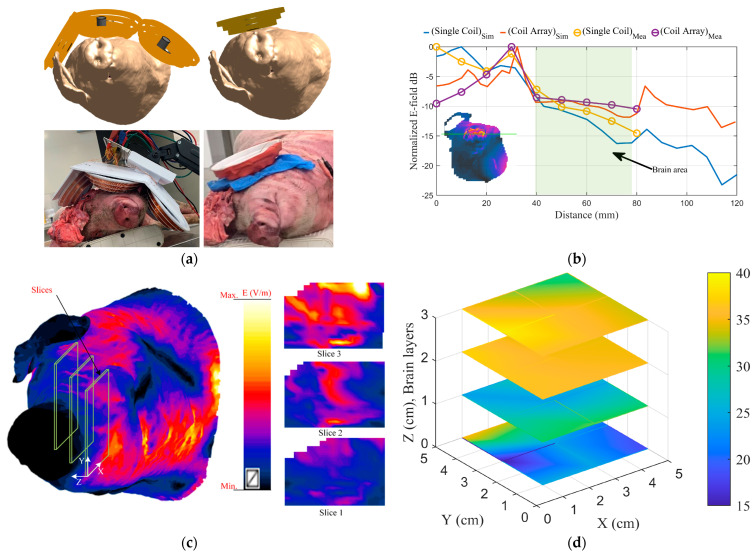
(**a**) The pig head with one cone coil and with the coil array in the simulation and measurement. (**b**) The simulation and measurement results for E field distribution on a test line passing through the coronal cross-section of the head using one coil and the coil array. (**c**) The simulation results for the distribution of the E-field on different slices of the head. (**d**) The measurement results for the distribution of the E-field on different slices of the head.

Since the shape and size of the pig brain differ from those of humans, it is difficult to reconcile the in vitro measurement results with simulations performed on a realistic human head model. Hence, a realistic high-resolution virtual pig model from the IT’IS ViZoo population, displayed in the top row of Figure 9a, is imported into the Sim4life simulations. The same experimental setup is replicated in the Sim4life simulation environment, and a numerical study of the field distribution inside the pig brain with the coil array system mounted on it, is performed. The simulation results are compared to the measured data to validate the system. Figure 9b depicts the simulation and measurement results for the E field distribution on a test line through a coronal cross-section of the pig head using a single coil and the coil array system. Additionally, Figure 9c,d illustrate the simulation and experimental data of the E-field value and distribution on several slices of the pig head.

## 5. Discussions

The proposed coil array can generate an electrical field distribution in the brain of a realistic head phantom that can reach the neuronal activation threshold in deep brain structures while keeping the superficial tissues safe. The simulation results are validated through a series of in vitro and postmortem experiments. Figure 8 and Figure 9 show a significant agreement between the numerical simulation and the in vitro and postmortem experimental data. These two figures demonstrate that the coil array is more effective than a single coil in deep areas.

Even though in vitro validation using homogenous head models is a feasible approach for studies comparing different coil designs, absolute values of the derived field distribution are inaccurate and untrustworthy. When comparing Figure 8b and Figure 9b, it is clear that variation in tissue properties inside the head has a considerable impact on coil field distribution that cannot be neglected.

Although numerous coils can be used for deep brain stimulation, the field may not be focused on deep targets. Magnetic stimulation is distinguished by the fact that the peak E is regularly seen in shallow regions [66]. Figure 4a depicts the field distribution at the grey matter for various coil configurations. The proposed system’s focality is reasonable when compared to other conventional coil geometries due to the cancellation in the magnetic field caused by wisely choosing the current direction for the coils, as illustrated in the figure.

The proposed coil array can stimulate deep brain areas successfully, even up to a distance of 5 to 6 cm from the surface of the head (refer to Figure 4b). However, the standard Fo8 coil can only create suprathreshold fields in a shallow region of up to 2.5 cm underneath the coil’s center [22]. The depth of penetration is determined by the intensity of the pulse generator with respect to the motor threshold. Simulation results suggest that providing pulses less than 220% of the maximum safe limit for superficial grey matter stimulation effectively activates the hippocampal region, which is located 4–6 cm away from the surface.

When the modeling and measurement results for single-coil and coil array topologies are compared, the coil array outperforms the single coil, as seen in Figure 8 and Figure 9. In the superficial area, the field value of a single coil is larger than that of a coil array; however, in deep brain areas, the array produces a higher field than the single coil. This is due to the placement of the array coils with respect to the head and the constructive addition of the fields at deep areas. 

The capacity of the proposed coil array to activate deep brain areas such as the hippocampus while remaining safe in other brain regions could have significant clinical ramifications. Many recent rTMS studies in Alzheimer’s and Parkinson’s disease patients found substantial health improvements when cortical targets (left and right motor and dorsolateral prefrontal cortex) were stimulated with a typical figure-of-eight coil [67,68,69]. Treatment using a non-invasive deep brain stimulation system, such as the suggested coil array, will allow for the simultaneous stimulation of several brain areas, including the targeted structure; the penetration depth can be adjusted by altering the stimulator power or varying the distance between the coils and the skull.

The envisioned deep-TMS coil array is a novel tool with promise for both research and therapeutic applications in mental and neurological illnesses linked with deep brain dysfunction. This coil array can be improved further by controlling coil activation using an inverter system linked to a control panel; this configuration will provide flexibility in activating single or multiple coils. In addition, employing the inverter’s circuit to perform the quick sequential activation of the coils one at a time will enhance system focality.

In this work, the core’s response to the application of an external magnetic field is considered linear, knowing that such an assumption is not valid for relatively high field amplitudes. The obtained results, however, can be valid for materials with high saturation values. To fully account for the core’s response, the core’s material hysteresis loop should be modeled. 

## 6. Conclusions

To overcome the rapid decay of the field inside the brain, a coil array that includes five cone coils was designed for deep brain stimulation. Our numerical study indicated that the magnetic core and shielding increase the electric field intensity and enhance focality but do not improve the field decay rate. However, the decay rate can be reduced by increasing coil size at the expense of focality. Thus, the array was optimized in terms of size, numbers, positions, and structure to ensure the safe and efficient stimulation of the deep brain region (the hippocampus). The designed array efficiently maintained an electric field attenuation rate to approach the stimulation threshold in deep areas while keeping the field in all other regions within safety limits. The array was tested numerically on realistic human head models and experimentally on a head phantom and a postmortem pig head. The numerical and experimental results validate the efficacy of the proposed array in non-invasive and repetitive deep brain stimulation. 

## Figures and Tables

**Table 1:** Design parameters of the coil.

Coil Geometry	First Coil	Second Coil	Third Coil
Outer Diameter (mm)	62	64	30
Inner Diameter (mm)	56	40	20
Height (mm)	10	10	10
Number of Turns	30	30	60
Wire Diameter (mm)	1.2	1.2	1.2
Number of Layers	10	10	10

## Data Availability

All the date are presented in the paper.
